# Ghanaian men and happiness: socioeconomic predictors

**DOI:** 10.1016/j.heliyon.2022.e09072

**Published:** 2022-03-08

**Authors:** Abdul Rauf Alhassan

**Affiliations:** Department of Surgery, Tamale Teaching Hospital, P.O. Box TL 16, Tamale, Ghana

**Keywords:** Happiness, Ghanaian, Men, Predictors, Prevalence

## Abstract

**Background:**

According to neoclassical economics, happiness is related to increasing prosperity, but today financial gain as the sole source of happiness is demised by many factors such as education, health, and means of transportation. An emotional state of happiness can disturb health outcomes.

**Aim:**

This study aimed to study happiness among Ghanaian men and their socioeconomic predictors.

**Methodology:**

A descriptive cross-sectional study design was used relying on data from Ghana Multiple Indicator Cluster Survey (MICS) 2017/18. Chi-square for two variables analysis and binary logistics for multiple variables analysis.

**Results:**

The national prevalence of estimated happiness among men recorded in this current study was 79.8% and overall estimated unhappiness was 20.2%. And predictors of happiness were age, higher educational attainment, regional association, ethnicity, body functional status, health insurance status, alcohol, and cigarette use.

**Conclusion:**

Over one-five Ghanaian men are unhappy. That is the issue of happiness among Ghanaian men is a major issue and there is the need for policy enforcement towards education promotion, poverty reduction, and minimizing the use of a substance such as alcohol and cigarette.

## 1 Introduction

The positivity of emotion that predicts quality of life is known as happiness [1]. And also According to Tatarkiewicz, happiness is defined as “a lasting, complete and justified satisfaction with life as a whole.’’ [2]. Happiness can mirror a person's fruitful emotional affiliation with their environment and personal fulfillment or hopefulness [3]. Meanwhile, earlier studies have revealed that the emotional state of happiness can disturb health outcomes, such as morbidity [3, 4]. On the world happiness index ranking, Ghana was ranked 89 out of 150 countries for the year 2020 [5].

According to neoclassical economics, happiness is related to increasing prosperity, but today financial gain as the sole source of happiness is demised by many factors such as education, health, and means of transportation [6]. Results from a global study, on income, higher education, and happiness, level of people happiness increases as at the early stages of economic development but happiness start to decline after an optimal point of happiness as economic development continues to progress. Also, attaining higher education such as secondary or tertiary education as compared to primary education was positively associated with happiness [7]. Also in an earlier study on happiness and health, there was a better prediction of happiness by subjective as compared to objective health and those with debilitating pain and urinary incontinence were likely to suffer low happiness [8].

The variation of happiness and age is not very clear and a study has indicated that there is a U-shape relation between happiness and age, with minimum happiness between 40 and 50 years of the lower curve [9]. This further indicated that happiness for men stop to increase around 70 years, 10 years earlier for females [9]. In many studies, ethnic composition and life satisfaction were significantly associated [10, 11, 12, 13]. In Addai et al. study, Akans were about 98% more likely to be happy as compared to other unstudied ethnic groups [13].

A recent two studies in Ghana by Alhassan and Anyinzaam-Adolipore indicated that alcohol use and cigarette smoking among men were associated with life satisfaction and happiness, happiness was less among those who used these substances [14, 15]. The focus of these two studies was on predictors of substance use and their psychological effects, unlike this current study which focused on socioeconomic predictors of happiness. Also, in earlier Ghanaian micro-level study predictors of happiness and life satisfaction included income, ethnicity, Southern origin, and social capital variables [13]. Their study was based on data from the 2005–2008 world values survey, unlike this current study which is based on more recent data 2017/18.

Apart from Addai et al., there are few studies on predictors of happiness among Ghanaians, meanwhile, on the world happiness index ranking, Ghana was ranked 89 out of 150 countries for the year 2020, and also from the happiness age U-shape theory men happiness stop to increase around 70 years, 10 years earlier for females [5, 9]. Therefore, this study aimed to study happiness among Ghanaian men and their socioeconomic predictors. A strong immune system is essential for general health and to research, being happy will help the immune system stay strong [16]. And identifying modifiable predictor factors will help recommend ways to improve happiness.

## 2 Materials and methods

A design of descriptive cross-sectional survey was used for this study relying on data from Ghana Multiple Indicator Cluster Survey (MICS) 2017/18. A survey that was conducted from October 2017 to January 2018 by Ghana Statistical Service with partnership from some Ghana organizations and ministries such as the Ministry of Health, Ministry of Education, Ministry of Sanitation and Water Resources, Ministry of Gender, Children and Social Protection, Ghana Health Service, and the Ghana Education Service. Technical support was from United Nations Children's Fund (UNICEF), with financial support from UNICEF, KOICA, UNDP, USAID, and the World Bank through the Statistics for Results Facility – Catalytic Fund (SRF-CF).

### 2.1 Sampling and data collection

Clusters that represented sampling units were randomly selected in the first stage. Stratification was used to select the place of residence (i.e., urban stratum or rural stratum). The clusters were randomly selected during the first stage. In the second stage, systematic sampling was used for households’ selection. Six questionnaires used for data collection in the survey were: Household questionnaire, Water Quality Testing Questionnaire, Questionnaire for Individual Women, Questionnaire for Individual Men, Questionnaire for Children under Five, and Questionnaire for Children Age 5–17. In this current study, data from the Questionnaire for Individual Men was used. Details of the methodology used for the survey are available in MICS 2017/18 report [17].

### 2.2 Statistical analysis

SPSS version 20 (IBM Corp., 2011, and NY) was used for data analysis. Study results were presented using frequencies and percentages in tables. The association between dependent and independent variables was done using chi-square and the binary logistic regression model was used to identify the predictor variables of men's happiness in Ghana. The study's statistical significance was set at a p-value of <0.05.

### 2.3 Ethical consideration

The MICS team of UNICEF released the dataset for this study. Ethical approval was not necessary for this study because it involved a secondary analysis of a dataset without publicity to the identity of the participants and their households.

### 2.4 Study variables

Dependent variable: The dependent variable was overall happiness. Respondents were asked, “Would you say you are [1] very happy [2], moderately happy [3], neither happy nor unhappy [4], slightly unhappy, or [5] very unhappy?” Those who answered very happy or slightly happy were classed as happy, while those who answered neither happy nor unhappy, somewhat unhappy or very unhappy were classified as unhappy.

Independent variables: They were the socioeconomic characteristics of the respondents. They are age, union status, educational level, residence, region, ethnicity, functional difficulties, health insurance status, wealth index quintile, alcohol use, and cigarette use.

## 3 Results

Most (56.2%) of the men were from rural residences and more (54.5%) were married. Most (12.8%) of the respondents were from the Ashanti region and with ethnicity, most (35.8%) of them were from the Akan tribe. About 37.9% of them had a JSS/JHS educational level or no education. Health insurance coverage was 43.0% among the respondents and 26.6% of them were in the poorest wealth index quintile. The majority (94.6%) of them had no functional difficulty. Alcohol ever use was 48.7% and cigarette ever use was 12.7% (Table 1).

**Table 1:** Socioeconomic characteristics of the respondents.

		Frequency	Percentage
Age group	15–24 years	2424	45.5%
	25–34 years	1285	24.1%
	35 years and above	1614	30.3%
Currently in union	Yes, currently married	1929	36.3%
	Yes, living with a partner	492	9.2%
	No, not in union	2899	54.5%
Man educational level	Pre-primary or none	696	13.1%
	Primary	767	14.4%
	JSS/JHS/Middle	2017	37.9%
	SSS/SHS/Secondary	1325	24.9%
	Higher	518	9.7%
Residence	Urban	2396	43.8%
	Rural	3080	56.2%
Region	Western	513	9.4%
	Central	436	8.0%
	Greater Accra	620	11.3%
	Volta	471	8.6%
	Eastern	540	9.9%
	Ashanti	701	12.8%
	Brong Ahafo	488	8.9%
	Northern	634	11.6%
	Upper east	479	8.7%
	Upper west	594	10.8%
Ethnicity	Akan	1958	35.8%
	GA/Dangme	387	7.1%
	Ewe	663	12.1%
	Guan	189	3.5%
	Gruma	230	4.2%
	Mole Dagbani	1349	24.6%
	Grusi	240	4.4%
	Mande	17	0.3%
	Other	441	8.1%
Functional difficulties (age 18–49 years)	Yes	231	5.4%
	No	4078	94.6%
Have health insurance	Yes	2287	43.0%
	No	3036	57.0%
Wealth index quintile	Poorest	1416	26.6%
	Second	878	16.5%
	Middle	931	17.5%
	Fourth	1006	18.9%
	Richest	1092	20.5%
Alcohol use	No	2729	51.3%
	Yes	2593	48.7%
ever smoke cigarette	No	4648	87.3%
	Yes	674	12.7%

### 3.1 Prevalence happiness among Ghanaian men and associated factors

The national prevalence of estimated happiness among men recorded in this current study was 79.8% and overall estimated unhappiness was 20.2%. In terms of life satisfaction, 15.1% think life satisfaction the previous year was worse and only 0.5% expected life satisfaction for the coming year to be worse (Table 2).

**Table 2:** Respondents life satisfaction and happiness.

		Frequency	Percentage
Estimation of overall happiness	Very happy	2236	42.0%
	Somewhat happy	2010	37.8%
	Neither happy nor unhappy	726	13.6%
	Somewhat unhappy	222	4.2%
	Very unhappy	129	2.4%
Life satisfaction compared to last year	Improved	3501	65.8%
	More or less than the same	1016	19.1%
	Worsen	802	15.1%
Life satisfaction expectation for next year	Improved	5160	97.2%
	More or less than the same	124	2.3%
	Worsen	24	0.5%

Chi-square analysis revealed a significant relationship (P ≤ 0.001), between overall happiness and socioeconomic characteristics such as age, marital status, region from, ethnicity, educational level, functional difficulties, health insurance coverage, wealth index quintile, alcohol, and cigarette use (Table 3).

**Table 3:** Relationship between socioeconomic characteristics and overall happiness.

		Overall life happiness		*X*^2^	df	P-value
		Unhappy	Happy			
Age group	15–24 years	399	2025	40.834	2	≤0.001
	25–34 years	287	998			
	35 years and above	391	1223			
Currently in union	Married	411	1518	40.971	2	≤0.001
	Co-habitation	148	344			
	Single	518	2381			
Man educational level	Pre-primary or none	162	534	23.711	4	≤0.001
	Primary	180	587			
	JSS/JHS/Middle	403	1614			
	SSS/SHS/Secondary	262	1063			
	Higher	70	448			
Residence	Urban	470	1866	.033	1	.856
	Rural	607	2380			
Region	Western	88	422	142.354	9	≤0.001
	Central	110	323			
	Greater Accra	110	491			
	Volta	130	325			
	Eastern	124	376			
	Ashanti	173	511			
	Brong Ahafo	116	356			
	Northern	127	493			
	Upper east	21	448			
	Upper west	78	501			
Ethnicity	Akan	418	1495	93.476	8	≤0.001
	GA/Dangme	88	284			
	Ewe	150	490			
	Guan	43	140			
	Gruma	74	153			
	Mole Dagbani	159	1159			
	Grusi	38	191			
	Mande	5	12			
	Other	101	321			
Functional difficulties (age 18–49 years)	Yes	88	143	36.578	1	≤0.001
	No	862	3216			
Have health insurance	Yes	354	1933	56.623	1	≤0.001
	No	723	2313			
Wealth index quintile	Poorest Second Middle Fourth Richest	281 196 228 216 156	1135 682 703 790 936	37.850	4	≤0.001
Alcohol use	No	443	2286	55.146	1	≤0.001
	Yes	634	1959			
ever smoke cigarette	No	882	3766	36.146	1	≤0.001
	Yes	195	479			

### 3.2 Socioeconomic predictors of happiness among Ghanaian men

Variables significant at the bivariate analysis level were further modeled with a binary logistic regression model to identify predictors. Age predicted happiness, men of age 35 years and above were less likely about 27% to be happy as compared to men of the age group of 15–24 years (AOR = 0.73, 95%, C.I. = 0.56–94). Higher educational attainment predicted happiness among Ghanaian men, men with higher education were about 56% more likely to be happy as compared to those without education (AOR = 1.56, 95%, C.I. = 1.07–2.28). Again the regional orientation of men predicted happiness among them, men from the Central region were less likely about 34% to be happy when compared with men from the Western region (AOR = 0.66, 95%, C.I. = 0.477–93). Those from the Volta region were likely about 44% less to enjoy happiness as compared to those from the western region (AOR = 0.56, 95%, C.I. = 0.37–0.85). Also, men from the Ashanti region were 43% less likely to be happy when compared to men from the Western region (AOR = 0.57, 95%, C.I. = 0.41–0.77). Again men from the Brong-Ahafo region were 32% less likely to be happy as compared to men from the Western region (AOR = 0.68, C.I. = 0.49–0.97). However, men from the Upper East region were almost 400% more likely to be happy as compared to men from the Western region (AOR = 4.93, 95%, C.I. = 2, 71–8.96). In terms of ethnicity men of the Gurma tribe were less likely about 49% to enjoy happiness when compared to those Akan tribe (AOR = 0.51, 95%, C.I. = 0.34–0.77). Those without functional difficulties were about 71% more likely enjoy happiness (AOR = 1.71, 95%, C.I. = 1.27–2.30). Those without health insurance were less likely about 36% to be happy (AOR = 0.74, 95%, C.I. = 0.63–0.88). Men from the richest wealth index quintile were more likely about 86% to be happy as compared to those from the poorest wealth index quintile (AOR = 1.86, 95%, C.I. = 1.37–2.53). The use of alcohol and cigarette protected from happiness, alcohol use predicted happiness less by 24% (AOR = 0.76, 95%, C.I. = 0.64–0.90) and cigarette use predicted happiness by 24% (AOR = 0.76, 95%, C.I. = 0.62–0.94) (Table 4).

**Table 4:** Binary logistic regression for socioeconomic predictors of life happiness.

	B	Wald	Sig.	AOR	95% C.I. for AOR	
					Lower	Upper
15–24 years		**Reference**	.049			
25–34 years	-.213	3.442	.064	.808	.645	1.012
35 years and above	-.320	5.935	**.015**	**.726**	**.561**	**.939**
Married		**Reference**	.337			
Co-habitation	-.141	1.210	.271	.868	.675	1.117
Single	-.158	1.844	.175	.853	.679	1.073
Pre-primary or none		**Reference**	.171			
Primary	.099	.436	.509	1.104	.823	1.481
JSS/JHS/Middle	.244	3.143	.076	1.277	.975	1.672
SSS/SHS/Secondary	.188	1.534	.216	1.206	.896	1.624
Higher	.446	5.313	**.021**	**1.562**	**1.069**	**2.282**
Western		**Reference**	.000			
Central	-.419	5.565	**.018**	**.658**	**.465**	**.932**
Greater Accra	-.142	.614	.433	.867	.608	1.238
Volta	-.584	7.486	**.006**	**.558**	**.367**	**.847**
Eastern	-.329	3.410	.065	.720	.508	1.020
Ashanti	-.572	12.664	**.000**	**.564**	**.412**	**.773**
Brong Ahafo	-.379	4.626	**.031**	**.684**	**.485**	**.967**
Northern	-.102	.252	.615	.903	.606	1.345
Upper east	1.594	27.309	**.000**	**4.925**	**2.709**	**8.956**
Upper west	.423	3.610	.057	1.526	.987	2.360
Akan		**Reference**	.000			
GA/Dangme	-.048	.092	.761	.953	.697	1.302
Ewe	.293	3.427	.064	1.341	.983	1.828
Guan	.384	2.532	.112	1.469	.915	2.358
Gruma	-.671	10.539	**.001**	**.511**	**.341**	**.766**
Mole Dagbani	.258	2.820	.093	1.295	.958	1.750
Grusi	-.040	.030	.864	.961	.610	1.514
Mande	-.504	.800	.371	.604	.200	1.823
Other	-.132	.694	.405	.876	.643	1.195
Functional difficulties (age 18–49 years) (No/Yes)	.535	12.380	**.000**	**1.708**	**1.267**	**2.300**
Health insurance (No/Yes)	-.295	11.630	**.001**	**.744**	**.628**	**.882**
Poorest		**Reference**	.000			
Second	.059	.215	.643	1.061	.826	1.363
Middle	-.068	.272	.602	.934	.724	1.206
Fourth	.063	.216	.642	1.065	.815	1.392
Richest	.621	15.811	**.000**	**1.861**	**1.370**	**2.528**
Alcohol use (Yes/No)	-.281	10.135	**.001**	**.755**	**.635**	**.898**
Ever smoke Cigarette (Yes/No)	-.270	6.744	**.009**	**.763**	**.622**	**.936**

## 4 Discussion

In terms of world happiness ranking Ghana was ranked 89 out of 150 countries for the year 2020 [5]. And in this current study, the national prevalence of estimated happiness among men was 79.8% and estimated unhappiness was 20.2%. In terms of life satisfaction, 15.1% think life satisfaction the previous year was worse and only 0.5% expected life satisfaction for the coming year to be worse. Meanwhile, the average happiness among Ghanaian from the duration of 2010–2019 was 5.7 out of 10 [18]. The implication from this current study is that men are generally happier compared to the general population.

Age predicted happiness, men of age 35 years and above were less likely about 27% to be happy as compared to men of the age group of 15–24 years. According to studies, there is a U-shape relation between happiness and age, with minimum happiness between 40 and 50 years of the lower curve [9, 19]. This current did confirmed that age group of 35 years and above was predictor of low happiness.

Also, higher educational attainment predicted happiness among Ghanaian men, men with higher education were about 56% more likely to be happy as compared to those without education. Similar to Din et al. study, attaining higher education such as secondary or tertiary education as compared to primary education was associated positively associated with happiness [7]. Also, 2018 panel data from 145 countries revealed a positive correlation between educational level and level of happiness [20].

Furthermore, the region of men predicted happiness among men, men from the Central region were less likely about 34% to be happy when compared with men from the Western region. Those from the Volta region were likely about 44% less to enjoy happiness as compared to those from the western region. Also, men from the Ashanti region were 43% less likely to be happy when compared to men from the Western region. Again men from the Brong-Ahafo region were 32% less likely to be happy as compared to men from the Western region. However, men from the Upper East region were almost 400% more likely to be happy as compared to men from the Western region. From Florida et al. study in USA metropolitan level of a region is related to housing affordability, therefore identity with the place of residence, and is associated with happiness [21].

Again, in terms of ethnicity men of the Gurma tribe were less likely about 49% to enjoy happiness when compared to those of Akan tribe. In many studies, ethnic composition and life satisfaction were significantly associated [10, 11, 12, 13]. Similar to this current study, in Addai et al. study, Akans were about 98% more likely to be happy as compared to other unstudied ethnic groups [13]. Ethnic inequality is one of America's most pressing societal challenges, and the observable characteristics differences in whites and blacks is associated with widing gap of happiness among them [22].

In an earlier study on happiness and health, there was a better prediction of happiness by subjective as compared to objective health and those with debilitating pain and urinary incontinence were likely to suffer low happiness [8]. And in this current study, those without functional difficulties were about 71% more likely to enjoy happiness and those without health insurance were less likely about 36% to be happy. On the vice versa emotional state of happiness can affect one health or morbidity [3, 4]. Also, a study in Russia and Ukraine concluded that health had greater influence on happiness [23].

According to neoclassical economics, happiness is related to increasing prosperity, but today financial gain as the sole source of happiness is demised by many factors such as education, health, and means of transportation [6]. Results from a global study, on income, higher education, and happiness, level of people happiness increases as at the early stages of economic development but happiness start to decline after an optimal point of happiness as economic development continues to progress [7]. And in this current study, men from the richest wealth index quintile were more likely about 86% to be happy as compared to those from the poorest wealth index quintile. This study finding is confirmation of global findings of wealth being predictor of happiness [7].

Finally, the use of alcohol and cigarette protected from happiness, alcohol and cigarette use predicted happiness by 24%. This current study result confirms results from earlier two studies by Alhassan et al. that substance such as alcohol and cigarette intake is associated with unhappiness [14, 15].

The limitation of this study was that not all factors related to the topic were explored since the study relied on secondary data for analysis and the result could be affected by social desirability bias.

## 5 Conclusion

Over one-five Ghanaian men are unhappy and and there is the need for policy enforcement towards higher education promotion, poverty reduction, and minimizing the use of a substance such as alcohol and cigarette.

## Declarations

### Author contribution statement

Abdul Rauf Alhassan: Conceived and designed the experiments; Performed the experiments; Analyzed and interpreted the data; Contributed reagents, materials, analysis tools or data; Wrote the paper.

### Funding statement

This research did not receive any specific grant from funding agencies in the public, commercial, or not-for-profit sectors.

### Data availability statement

Data associated with this study has been deposited at https://mics.unicef.org.

### Declaration of interests statement

The authors declare no conflict of interest.

### Additional information

No additional information is available for this paper.
